# Simultaneous optimization of the acidified water extraction for total anthocyanin content, total phenolic content, and antioxidant activity of blue honeysuckle berries (*Lonicera caerulea* L.) using response surface methodology

**DOI:** 10.1002/fsn3.1152

**Published:** 2019-08-10

**Authors:** Fengfeng Li, Hengtian Zhao, Ruiru Xu, Xiuling Zhang, Wentao Zhang, Meiling Du, Xiaochen Liu, Lili Fan

**Affiliations:** ^1^ College of Food Science Northeast Agriculture University Harbin China; ^2^ Northeast Institute of Geography and Agroecology Chinese Academy of Sciences Harbin China

**Keywords:** antioxidant activity, blue honeysuckle berries (*Lonicera caerulea* L.), Box–Behnken design, response surface methodology, total anthocyanin content, total phenolic content

## Abstract

The purpose of this study was to optimize the total anthocyanin content (TAC), total phenolic content (TPC), and antioxidant activity of acidified water extract from blue honeysuckle berries by response surface methodology (RSM). The optimized conditions were HCl concentration of 0.35%, liquid–solid ratio of 49.42 ml/g, and extraction temperature of 41.56°C for total anthocyanin content (24.01 ± 0.37 mg/g), total phenolic content (207.03 ± 3.31 mg/g), DPPH radical scavenging activity (68.24 ± 1.13%), and ABTS radical scavenging activity (70.05 ± 0.84%). The experimental results are consistent with the predicted values. The results showed that acidified water extraction was an effective, simple, and green technique for the extraction of total anthocyanins, total phenol, and antioxidant activity from blue honeysuckle berries.

## 1 INTRODUCTION

Wild edible berries are receiving increasing attention due to their health benefits. This is that they contain large amounts of ascorbic acid, phenolics, and anthocyanins (Cosmulescu, Trandafir, & Nour, 2017). Research of anthocyanins and polyphenols has been focused on due to their numerous biological activities, such as antioxidation, antibacterial, antitumor, antidiabetes, and anti‐inflammatory (Li, Yu, Zhang, Ke, & Hong, 2018; Misbah, Aziz, & Aminudin, 2013; Mizgier et al., 2016; Puupponen‐Pimiä et al., 2010).

Blue honeysuckle berries (*Lonicera caerulea* L.) are a new wild berry resource belong to Caprifoliaceae family. They are mostly distributed in northeastern Russia, China, and Japan. They are usually used for making juice, jam, wine, puffed snacks, food‐coloring agents, and dietary supplements (Liu et al., 2010; Jan, O., Aneta, W., & Sabina, L., 2016). Currently, blue honeysuckle berries are becoming ever more popular, mainly because they promote human health. Blue honeysuckle berry extract has various bioactivities, including antioxidation antibacterial, anti‐inflammatory, antiproliferative, and lowering cholesterol properties (Caprioli et al., 2016; Celli, Ghanem, & Brooks, 2014; Jin et al., 2006; Jurikova et al., 2014; Liu, Wu, Guo, Meng, & Chang, 2018; Raudseppab et al., 2013; Rupasinghe, Yu, Bhullar, & BorsBob, 2012; Wang et al., 2017; Wu et al., 2015). These biological activities may be due to their high levels of vitamin C and polyphonic compounds, particularly anthocyanins (cyanidin‐3‐glucoside as standard; Chaovanalikit, Thompson, & Wrolstad, 2004; Heinrich et al., 2013; Wojdyło, Jáuregui, CarbonellBarrachina, Oszmiański, & Golis, 2013). The extraction technology of anthocyanins in blue honeysuckle berries has been investigated. For example, Giovana Bonat C (Giovana Bonat, Amyl, & Marianne Su‐Ling, 2015) studied the optimization of ultrasound‐assisted extraction of anthocyanins from haskap berries. Liu S (Liu et al., 2016) developed optimum for high hydrostatic pressure on anthocyanin extracts of wild *L caerulea* berry. However, studies on the simultaneous extraction of anthocyanin, polyphenol, and antioxidant activities from *L japonica* berries have not been reported.

Extraction is the first stage in the utilization and further study of bioactive compounds. The extraction rate of anthocyanins and polyphenols was affected by various factors, including the extraction method, solvent, temperature, time, and pH (Blackhall, Berry, Davies, & Walls, 2018; Ćujić et al., 2016; Maran, Manikandan, Nivetha, & Dinesh, 2017). The most commonly used extraction solvent is acidified ethanol. However, due to the toxicity of organic solvents, people worry that they may be retained in extracts and thus have an impact on human health. Therefore, reducing the consumption of organic solvents can provide safer and more reliable extracts. This has been applied in the extraction phenolic compounds from black soybeans (Ryu & Koh, 2018).

RSM is a statistical method for solving multivariable and its interaction by using reasonable experimental design (Bezerra, Santelli, Oliveira, Villar, & Escaleira, 2008). RSM has been applied to optimize the extraction of polyphenols in various plants, such as *Lycium ruthenicum Murr.* (Chen et al., 2018), *Berberis asiatica* fruits (Belwal, Dhyani, Bhatt, Rawal, & Pande, 2016), Feijoa sellowiana leaves (Poodi et al., 2018), and *Rheum moorcroftianum* (Pandey, Belwal, Sekar, Bhatt, & Rawal, 2018), and they were found successful in predicting the model.

In this study, Box–Behnken design (BBD) was used for the first time to optimize the acidified water extraction process of TAC and Total phenolic content (TPC) from blue honeysuckle berries and to determine their antioxidant activities. The different independent variables, including the hydrochloric acid (HCl) concentration (*X*_1_), the solid–liquid ratio (*X*_2_), and extraction temperature (*X*_3_), and their interactions were studied by RSM. This study provides an economical and feasible method for extracting anthocyanin from blue honeysuckle berries and excavates the value of blue honeysuckle berries, which can be further used in the industrial application and pharmacological activity analysis.

## 2 MATERIALS AND METHODS

### 2.1 Materials

Ripe wild blue honeysuckle berries were purchased from a local market in Yichun, Heilongjiang Province, China, in June 2017. Berries were washed with tap water to remove surface dirt. The stems, leaves, and stones were removed manually. Berries were preserved at −20°C for 24 hr and then freeze‐dried. Frozen berries were dried by vacuum freeze (LGJ‐1A‐50, Yatai Cologne Instrument Technology Co., Ltd) until a constant weight is gained. Dried berries were grounded into powder using a mill (LFP‐100, Shanghai Phillips Industries Co., Ltd) and passed through a 60‐mesh sieve. Before experimental analysis, the powdered samples were sealed and stored at 4°C.

### 2.2 Chemicals and reagents

Folin–Ciocalteu's reagent, gallic acid, rutin, 2,2‐diphenyl‐1‐picrylhydrazyl (DPPH), and 2,2‐azino‐bis‐(3‐ethylbenzothiazoline‐6‐sulfonic acid)‐diammonium salt (ABTS) were purchased from Sigma‐Aldrich. Hydrochloric acid, potassium chloride, hydrochloric acid, sodium acetate, sodium carbonate, sodium nitrite, aluminum chloride, sodium hydroxide, potassium persulphate, methanol, and ethanol were obtained from general suppliers.

### 2.3 Selection of variables

The effects of different variables such as liquid–solid ratio, pH, extraction time, and temperature were known to affect anthocyanin extraction yield. The right range of each factor should be chosen, and HCl concentration (0.1%–0.5%), the liquid–solid ratio (20–60 ml/g), extraction time (30–150 min), and temperature (20–60°C) were initially applied as single factors. Blue honeysuckle berries were invoked as test materials, and anthocyanin was extracted from each individual index under diverse conditions, while the extraction conditions of the other three indicators remained unchanged.

### 2.4 Box–Behnken design for extraction optimization

Optimization experiments for total anthocyanin content, TPC, and antioxidant activities in blue honeysuckle berries were performed. The combined variable conditions were determined by using a three‐level three‐factor BBD. These three independent variables were encoded in three levels (−1, 0, and +1; Table 1). The results were fitted using a response surface regression to fit the quadratic polynomial equation as follows:

(1) $$Y=\beta_{0}+\sum_{i=1}^{k}\beta_{i}X_{i}+\sum_{i=1}^{k}\beta_{\mathrm{ii}}X_{\mathrm{ii}}^{2}+\sum_{i}^{k-1}\sum_{j}^{k}\beta_{\mathrm{ij}}X_{i}X_{j}$$

where *Y* is the response variables (TA, TP); *X_i_* and *X_j_* are independent variables. *β*_0_ is the constant coefficient; *β_i_* is the linear coefficient; *β_ii_* is the quadratic coefficient; and *β_ij_* is the cross product coefficients.

**Table 1:** Box–Behnken design (BBD) for the independent variables and corresponding response values

Run	Extraction conditions			Experimental results			
	HCl concentration (%) (*X*_1_)	Liquid–solid ratio (ml/ g) (*X*_2_)	Temperature (°C) (*X*_3_)	TAC (mg/g)	TPC (mg GAE/g)	DPPH radical scavenging activity (%)	ABTS radical scavenging activity (%)
1	0.3 (−1)	50 (0)	30 (−1)	22.65	195.74	73.56	61.36
2	0.4 (0)	40 (−1)	30 (−1)	23.19	184.02	69.10	69.27
3	0.5 (1)	50 (0)	30 (−1)	22.60	192.04	69.97	55.05
4	0.4 (0)	60 (1)	30 (−1)	25.34	204.16	67.91	55.11
5	0.5 (1)	60 (1)	40 (0)	24.43	203.91	68.23	57.76
6	0.3 (−1)	60 (1)	40 (0)	24.65	210.26	74.46	60.26
7	0.3 (−1)	40 (−1)	40 (0)	23.70	187.73	72.83	76.01
8	0.5 (1)	40 (−1)	40 (0)	23.74	187.69	68.06	72.08
9	0.4 (0)	60 (1)	50 (1)	25.25	214.12	67.21	58.33
10	0.3 (−1)	50 (0)	50 (1)	22.23	201.06	73.54	68.75
11	0.4 (0)	40 (−1)	50 (1)	22.93	186.90	67.80	73.91
12	0.5 (1)	50 (0)	50 (1)	22.19	208.06	66.49	63.91
13	0.4 (0)	50 (0)	40 (0)	25.68	213.96	69.87	65.31
14	0.4 (0)	50 (0)	40 (0)	25.37	216.69	69.83	65.94
15	0.4 (0)	50 (0)	40 (0)	25.85	208.98	69.57	66.41
16	0.4 (0)	50 (0)	40 (0)	26.68	212.10	71.30	67.97
17	0.4 (0)	50 (0)	40 (0)	25.74	209.15	69.74	66.72

ANOVA was performed using Design Expert 8.0.6 Trial (Stat‐Ease, Inc.) to determine the linear, quadratic, and interacting regression coefficients (*β*) of the individual. The adaptability of a polynomial equation in response was estimated by using the decision coefficient (*R* ^2^), and the significance of dependent variables was analyzed statistically by calculating *F* value (*p* < .05).

### 2.5 Sample extraction

Blue honeysuckle berry powdered sample (1 g) was mixed with distilled water of different volumes (30–50 ml) and different HCl concentrations (0.3%–0.5%) in a 100‐ml triangular flask. Extraction of mixture by oscillating water bath (HH‐4, Sepp Experimental Instrument Factory) at various temperatures (30–70°C) for different time periods (30–150 min). The extract was filtered through a qualitative filter paper and stored at 4°C before the experimental process. All experiments were conducted in parallel three times.

### 2.6 Total anthocyanin content

Total anthocyanin content was measured by the pH differential method (He et al., 2016). Diluting an aliquot of with potassium chloride buffer (0.025 M, pH 1.0) and sodium acetate buffer (0.4 M, pH 4.5) in a 50‐ml volumetric flask and allowed to equilibrate for 1 hr. Distilled water was used as blank, and the absorbance was recorded at 530 nm and 700 nm with a spectrophotometer. Total anthocyanin concentration in the extract was calculated using the following equation:

(2) $$\text{TAC}\;\left(\text{mg/g}\right)=\frac{A\times MW\times DF\times V\times 1000}{\varepsilon \times m\times 1}$$

where *A* is pH 1.0 (A530 nm − A700 nm) − pH 4.5 (A530 nm − A700 nm), MW is the molecular weight of cyanidin‐3‐glucoside (449.2 g/mol), DF is the dilution factor, *ε* is the molar extinction coefficient of cyanidin‐3‐glucoside (26,900 L/mol × cm), 1 is for a standard 1 cm path length, m is the quantity of sample (g), and *V* is the total volume (ml). Total anthocyanin content was expressed as mg cyanidin‐3‐O‐glucoside equivalents per g blue honeysuckle.

### 2.7 Total phenolic content

Total phenolic content was determined by the Folin–Ciocalteu method following Klavins, Kviesis, Nakurte, and Klavins (2018) with minor modifications. Briefly, 0.5 ml sample was mixed with 2.5 ml of Folin–Ciocalteu reagent (0.2 mol/L), and 2.0 ml of sodium carbonate (7.5 g/100 ml) was inserted after 5 min. After incubation at room temperature and darkness for 2 hr, the absorbance of the mixture to the reagent blank solution (0.5 ml distilled water instead of the sample) was measured at 760 nm by spectrophotometer. The results were calculated according to the calibration curve of gallic acid and expressed as gallic acid equivalents (mg GAE/100 g). All samples were analyzed for three times, and then, the average value was taken.

### 2.8 Determination of DPPH scavenging activity

Radical scavenging activity of DPPH (1, 1‐ two phenyl‐2‐pyridinium hydrazide) was determined by an improved method (Heinrich et al., 2013). DPPH solution (0.1 mm) was prepared in ethanol. 2 ml sample extract was added to 2 ml of DPPH reagent. The reaction was carried out for 30 min at room temperature in the dark, and the absorbance was measured using a spectrophotometer at 517 nm. Inhibition percent of scavenged DPPH was calculated as 100× (A_b_ − A_s_)/A_b_, where A_b_ is the absorbance of the blank and As is the absorbance of the sample.

### 2.9 Determination of ABTS+ scavenging activity

ABTS radical scavenging capacity was performed according to the method displayed by Xu (Xu et al., 2017) previously. ABTS mother liquid was prepared by the reaction of 7 mM ABTS solution and an equal amount of 2.45 mM potassium persulfate solution. The mixture was incubated in darkness at room temperature for 16 hr and then diluted with 0.1 mM phosphate buffer (pH 7.0) to an absorbance of 0.7 ± 0.02 at 734 nm. Prepared 3.9 ml ABTS solution was mixed with differently diluted blue honeysuckle berry extract at 0.1 ml and then stored for 10 min at room temperature in the dark. Absorbance was recorded in 734 nm. Ethanol was used instead of the extract for the blank test. Inhibition percent of scavenged ABTS was calculated as 100 × (A_b_ − A_s_)/A_b_, where A_b_ is the absorbance of the blank and As is the absorbance of the sample.

### 2.10 Verification of the model

The optimized conditions were validated for the maximum phenolic compounds (TAC, TPC) and antioxidant activities (DPPH, ABTS) based on the values obtained using RSM. All the responses were determined under optimized conditions of the extraction. The experimental values were compared with predicted values based on CV% in order to determine the validity of the model.

## 3 RESULTS AND DISCUSSION

### 3.1 Results of selection of extraction parameters

**Figure 1:**
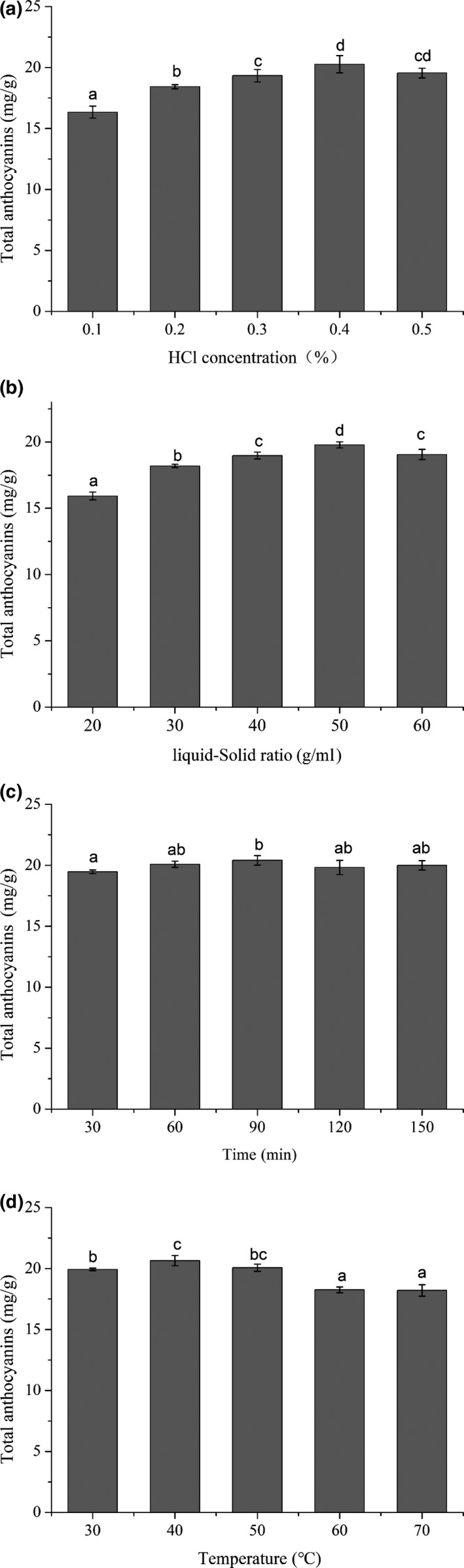
(a) Effects of HCl concentration on total anthocyanin content. (b) Effects of liquid–solid ratio on total anthocyanin content. (c) Effects of time on total anthocyanin content. (d) Effects of temperature on total anthocyanin content

According to the previous studies, we know that extraction time, temperature, pH value, liquid–solid ratio, and other factors have a significant impact on the extraction rate of anthocyanins (Ćujić et al., 2016; Li et al., 2017). Extraction of total anthocyanin from blue honeysuckle berries was studied by choosing four key parameters: concentration of HCl (pH), liquid–solid ratio, extraction time, and extraction temperature. It can be seen from Figure 1 that the concentration of HCl, liquid–solid ratio, and extraction temperature have significant effects on the extraction rate of total anthocyanins from blue honeysuckle berries, except the extraction time. As shown in Figure 1a, the total anthocyanin content increased significantly between 0.1% and 0.4% of HCl concentration, and the total anthocyanin value above the concentration began to decrease. Similar findings have been found in studies on the extraction of anthocyanins from black soybeans (Blackhall et al., 2018). Figure 1b shows the influence of liquid‐to‐solid ratio on anthocyanin extraction yield. The extraction amount increased significantly from 20 to 50 and then decreased slightly at 60. The results show that, within a certain range, the higher the solid‐solvent ratio, the higher the amount of anthocyanins. As shown in Figure 1c, anthocyanin value increases gradually between 30 and 40°C. However, when the extraction temperature further increased above 40°C, the anthocyanin value gradually decreased. This is probably because the diffusion and mass transfer of the solvent increases as the extraction temperature increases. However, too high a temperature will destroy the anthocyanin component of the extract. Interestingly, the extraction time had no significant effect on the total anthocyanin content of 30–150 min. Through the above experiments, the RSM design chooses HCl concentration (0.3%–0.5%), liquid–solid ratio (40–60 ml/g), and extraction temperature (30–50°C) as the extraction parameters.

### 3.2 Fitting the model

The BBD in the optimization experiment consisted of four factors; three levels and five center point runs were carried out in triplicate. The experimental conditions and results of 17 runs are given in Table 1. Regression coefficients, coefficient of determination (*R* ^2^), and *F* values for the dependent variables are presented in Table 2.

**Table 2:** Regression coefficient (*β*), coefficient of determination (*R* ^2^), and *F* test value of the predicted second‐order polynomial models for the phenolic compounds and antioxidant activities

Regression coefficients (*β*)				
	TAC	TPC	DPPH	ABTS
Intercept, *X*_0_	25.86	212.18	70.07	66.47
Linear				
*X*_1_	−0.034	−1.57	−2.71***	−2.2**
*X*_2_	0.76**	6.65**	2.500E‐003	−7.48***
*X*_3_	−0.15	4.27*	0.69*	3.01***
Quadratic				
$X_{1}^{2}$	−1.75***	−14.65***	1.86**	−0.92
$X_{2}^{2}$	0.013	−0.13	−1.03*	0.97
$X_{3}^{2}$	−1.70***	−6.52*	−1.03*	−3.29**
Cross product				
*X*_1_ *X*_2_	−0.065	−1.58	−0.36	0.36
*X*_1_ *X*_3_	2.500E‐003	−5.55*	−0.87	0.37
*X*_2_ *X*_3_	0.043	−0.59	0.15	−0.36
*R* ^2^	0.9404	0.9808	0.9582	0.9753
*F* value (model)	12.27**	10.70**	17.83***	30.67***
*F* value (lack of fit)	1.43	2.70	1.30	3.92

Abbreviations: ABTS, 2,2‐azino‐bis‐(3‐ethylbenzothiazoline‐6‐sulfonic acid)‐diammonium salt; DPPH, 2,2‐diphenyl‐1‐picrylhydrazyl; *R*_2_, coefficient of determination; TAC, Total Anthocyanin Content; TPC, Total Phenolic Content; *X*_1_, HCl concentration (%); *X*_2_, solid–liquid ratio (g/mL); *X*_3_, temperature (°C). Level of significance:

* *p* < .05,

** *p* < .01,

*** *p* < .001.

### 3.3 Total anthocyanin content

In Table 2, the ANOVA results showed the linear effects of liquid–solid ratio (*X*_2_), and the quadratic effects of $X_{1}^{2}$ and $X_{3}^{2}$ demonstrated significant effects on TAC. Based on the regression coefficient (*β*) values, the $X_{1}^{2}$ revealed a major effect, which was followed by $X_{3}^{2}$ and *X*_2_. The nonsignificant factors were removed, and the fitted second‐order polynomial equation was as follows:

(3) $$Y_{\text{TAC}}=25.86+0.76X_{2}-1.75X_{1}^{2}-1.70X_{3}^{2}$$

The nonsignificant value of lack of fit (*F* = 1.43) showed the model is fitted to the spatial influence of the variables in the response with good prediction (*R* ^2^ = .9404; Table 2).

**Figure 2:**
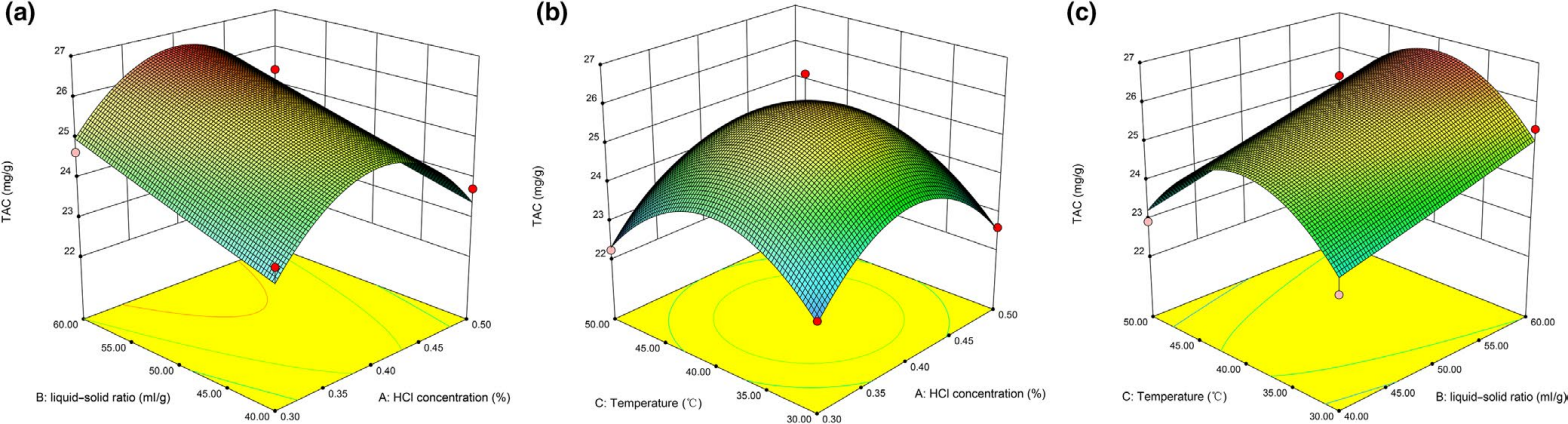
Response surface plot showing the effects of extraction variables on total anthocyanin content

The liquid–solid ratio (*X*_2_) showed a significant (*p* < .01) positive effect on TAC, while the quadratic effects of $X_{1}^{2}$ and $X_{3}^{2}$ had a significant (*p* < .001) negative effect on TA (Table 2). The TAC increased with an increasing solvent to the liquid–solid ratio (Figure 2). A proper increase in the liquid–solid ratio may be due to the increase in solvent dissolution of more solutes. This is in agreement with previous studies (Bezerra et al., 2008; Chen et al., 2018).

### 3.4 Total phenolic content

As shown in Table 2, the liquid–solid ratio (*X*_2_) and extraction temperature (*X*_3_) had a significant positive effect on TPC, while the quadratic ($X_{1}^{2}$, $X_{3}^{2}$) and the interaction(*X*_1_ *X*_3_) displayed a highly significant negative effect on TPC. TPC is more dependent on $X_{1}^{2}$, followed by *X*_2_, $X_{3}^{2}$, *X*_1_ *X*_3_, and *X*_3_. The nonsignificant factors were removed, and the fitted second‐order polynomial equation was as follows:

(4) $$Y_{\text{TPC}}=212.18+6.65X_{2}+4.27X_{3}-14.65X_{1}^{2}-6.52X_{3}^{2}-5.55X_{1}X_{3}$$

The nonsignificant value of lack of fit (*F* = 2.70) showed the model is fitted to the influence of the variables in the response with good prediction (*R* ^2^ = .9808; Table 2).

**Figure 3:**
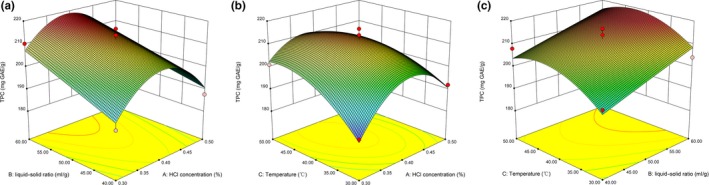
Response surface plot showing the effects of extraction variables on total phenolic content

The interaction between the HCl concentration and extraction temperature (*X*_1_ *X*_3_) showed a significant (*p* < .05) negative effect on TPC (Table 2). At lower HCl concentration, the extraction rate of TPC built up over with the increase in extraction temperature. However, over a higher HCl concentration, TPC decreased with increasing extraction temperature (Figure 3). This result is different from the previous study (Blackhall et al., 2018), which is due to the different varieties used in the experiment.

### 3.5 Effect of extraction variables on antioxidant activity

The antioxidant activity was determined by DPPH and ABTS methods. DPPH radical scavenging activity is primarily affected by X_1_, followed by $X_{1}^{2}$, $X_{2}^{2}$, $X_{3}^{2}$, and *X*_3_. ABTS radical scavenging activity is largely dependent on *X*_2_, followed by $X_{3}^{2}$, *X*_3_, and *X*_1_. The nonsignificant factors were removed, and the second‐order polynomial equations (DPPH and ABTS) of antioxidant activity were fitted. The results are as follows:

(5) $$Y_{\text{DPPH}}=70.07-2.71X_{1}+0.69X_{3}+1.86X_{1}^{2}-1.03_{2}^{2}-1.03X_{3}^{2}$$

(6) $$Y_{\text{ABTS}}=66.47-2.2X_{1}-7.48X_{2}+3.01X_{3}-3.29X_{3}^{2}$$

Lack of fitting values (*F* = 17.83 and 30.67) indicates that the models were fit with good prediction (*R* ^2^ = 0.9582 and 0.9753; Table 2)

**Figure 4:**
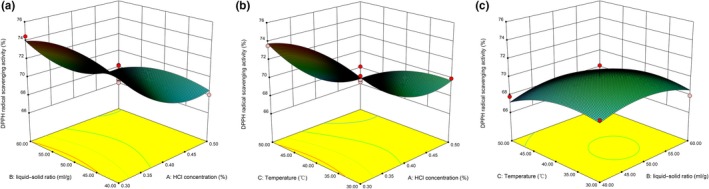
Response surface plot showing the effects of extraction variables on 2,2‐diphenyl‐1‐picrylhydrazyl radical scavenging activity

**Figure 5:**
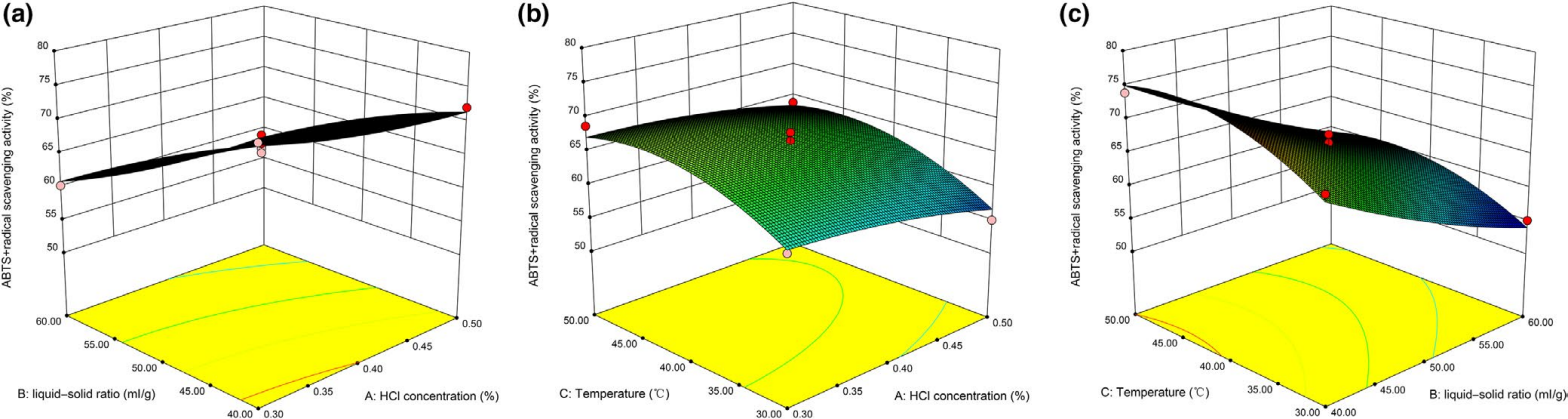
Response surface plot showing the effects of extraction variables on 2,2‐azino‐bis‐(3‐ethylbenzothiazoline‐6‐sulfonic acid)‐diammonium salt radical scavenging activity

In conclusion, the HCl concentration had a negative effect, meaning that lower HCl concentrations in solvent led to higher DPPH and ABTS scavenging activity. For the extraction temperature, it has a positive impact on DPPH and ABTS. Compared with DPPH, the liquid–solid ratio has a significant effect on ABTS. Similar results have been found in other studies, such as Blackhall et al., (2018). These results are shown in Figure 4 and Figure 5.

### 3.6 Model validation

The optimum conditions for the determination of TAC, TPC, and antioxidant activity (DPPH and ABTS) by RSM model were as follows: HCl concentration (0.35%), liquid–solid ratio (49.42 ml/g), and extraction temperature (41.56°C). Each group was tested three times in parallel, and the results were presented in Table 3. This fully demonstrates that the experimental value is quite close to the predicted value (the difference is less than 5%), and the validity and adequacy of the prediction model are demonstrated.

**Table 3:** Experiment data of the validation of predicted value at optimal extraction conditions

Dependent variables	Experimental value	Predicted value	Std. error
TAC (mg/g)	25.01 ± 0.37	25.42	1.61
TPC (mg GAE/g)	207.03 ± 3.31	210.32	1.56
DPPH (%)	68.74 ± 1.13	70.92	3.07
ABTS (%)	70.05 ± 0.84	69.41	0.92

Note

Std. error was calculated by comparing the experimental value and predicted value.

## 4 CONCLUSION

In the present study, the total anthocyanin content, TPC, and antioxidant activity of blue honeysuckle berries extracted by acidified water were optimized by RSM. Experimental conditions for maximum extraction rate were as follows: HCl concentration of 0.35%, the liquid–solid ratio of 49.42 ml/g, and an extraction temperature of 41.56°C. Under the optimum conditions, total anthocyanin content was (24.01 ± 0.37 mg/g), TPC was (207.03 ± 3.31 mg/g), DPPH radical scavenging activity was (68.24 ± 1.13%), and ABTS radical scavenging activity was  (70.05 ± 0.84%). The experimental results of the optimized are consistent with the predicted values. This acidified water extract can be considered as an easy and environmental tool for the extraction of both anthocyanins and total phenolic compounds from blue honeysuckle berries.

## CONFLICT OF INTEREST

The authors declare that they do not have any conflict of interest.

## ETHICAL APPROVAL

This study does not involve any human or animal testing.
